# Nitrogen-doped carbon nanotubes coated with zinc oxide nanoparticles as sulfur encapsulator for high-performance lithium/sulfur batteries

**DOI:** 10.3762/bjnano.9.159

**Published:** 2018-06-06

**Authors:** Yan Zhao, Zhengjun Liu, Liancheng Sun, Yongguang Zhang, Yuting Feng, Xin Wang, Indira Kurmanbayeva, Zhumabay Bakenov

**Affiliations:** 1 School of Materials Science & Engineering, Research Institute for Energy Equipment Materials, Hebei University of Technology, Tianjin 300130, China; 2 Synergy Innovation Institute of GDUT, Heyuan, Guangdong Province, China; 3 International Academy of Optoelectronics at Zhaoqing, South China Normal University, China; 4 Institute of Batteries LLC, National Laboratory Astana, Nazarbayev University, 53 Kabanbay Batyr Avenue, Astana 010000, Kazakhstan

**Keywords:** batteries, nanocomposites, sol–gel processes, sulfur, zinc oxide (ZnO)

## Abstract

Nitrogen-doped carbon nanotubes coated with zinc oxide nanoparticles (ZnO@NCNT) were prepared via a sol–gel route as sulfur encapsulator for lithium/sulfur (Li/S) batteries. The electrochemical properties of the S/ZnO@NCNT composite cathode were evaluated in Li/S batteries. It delivered an initial capacity of 1032 mAh·g^−1^ at a charge/discharge rate of 0.2C and maintained a reversible capacity of 665 mAh·g^−1^ after 100 cycles. The coulombic efficiency of the cathode remains unchanged above 99%, showing stable cycling performance. X-ray photoelectron spectroscopy analysis confirmed the formation of S–Zn and S–O bonds in the composite. This indicates that an enhanced cycling and rate capability of the S/ZnO@NCNT composite could be ascribed to advantages of the ZnO@NCNT matrix. In the composite, the active ZnO-rich surfaces offer a high sulfur-bonding capability and the NCNT core acts as a conductive framework providing pathways for ion and electron transport. The as-prepared S/ZnO@NCNT composite is a promising cathode material for Li/S batteries.

## Introduction

Due to its high theoretical specific capacity of 1672 mAh·g^−1^ and energy density of 2600 Wh·kg^−1^, sulfur has been considered as a promising cathode material for lithium/sulfur (Li/S) batteries [1]. Additionally, sulfur is naturally abundant, has low cost and is environmentally friendly. But it is not conductive, and it dissolves into the electrolyte in the form of lithium polysulfides (Li_2_S*_n_*, 4 ≤ *n* ≤ 8) during battery operation [2]. This is one of the major challenges in the commercialization of Li/S batteries. To overcome this problem, a rational design of the sulfur-based cathode is required, such as the addition of porous conductive materials that could “attract” or ”confine” the S atoms in the cathode, and, therefore, reduce any losses of S.

As an excellent conductive agent, carbon-based materials, e.g., carbon black, graphene and carbon nanotubes (CNTs), have been widely used in Li/S composite cathode materials [3]. In addition, by doping with N and a precise control of its morphology, these carbon materials can also play an active role in S confinement [4]. For example, it has been reported that the functional nitrogen groups in N-doped graphene (NG) sheets have a good binding capability for lithium polysulfides, which can greatly enhance the life of Li/S batteries [5]. Another popular strategy to reduce polysulfides from dissolution is using metal oxides, such as TiO_2_ [6], ZnO [7], MnO_2_ [8], and SiO_2_ [9], as the additives or coating layer in the S-cathode. This is because metal oxides can provide strong binding sites with S and reduce the shuttling effect [10]. In addition, metal oxides can be easily synthesized in various morphologies, e.g., hollow structures, to “hold” S [11]. Similar to S, metal oxides are, however, not conductive [12]. Therefore, an efficient approach is to use hybrids/composites of carbon materials and metal oxides, as they could provide strong binding sites to sulfur while simultaneously improving the conductivity of the electrode.

We previously reported the synthesis of ZnO nanoparticles on NCNT as anode material for Li-ion batteries [13], and focused on the effect of NCNT on ZnO nanoparticles. A high concentration of nucleation sites in NCNT allows ZnO to uniformly grow on its surface with a small size. Also, NCNT has a higher electrical conductivity due to its additional free electron pairs compared to CNT without nitrogen doping. The ZnO@NCNT composite showed excellent electrochemical properties in lithium-ion batteries with a reversible capacity of 664 mAh*·*g^−1^ after 100 cycles at a current density of 100 mA·g^−1^. Inspired by these results, we decided to use the ZnO@NCNT composite as a part of cathode in Li/S batteries, focusing on the effect of ZnO@NCNT on the absorption of polysulfides.

Accordingly, in this work, we synthesized nanocomposites of zinc oxide-coated nitrogen-doped carbon nanotubes with sulfur (S/ZnO@NCNT). ZnO was chosen because it is cheap, non-toxic and stable [14–15]. More importantly, ZnO demonstrates a strong affinity to polysulfides. In addition, NCNT was used due to its good conductivity and the ability of active nitrogen sites to enhance the electrochemical performances of Li/S batteries [13,16]. To the best of our knowledge, such uniquely structured S/ZnO@NCNT composites have been rarely reported as cathode material for Li/S battery in the literature. Here, we present a composite S/ZnO@NCNT cathode exhibiting stable performance and its structural and electrochemical analysis and evaluation.

## Results and Discussion

**Figure 1:**
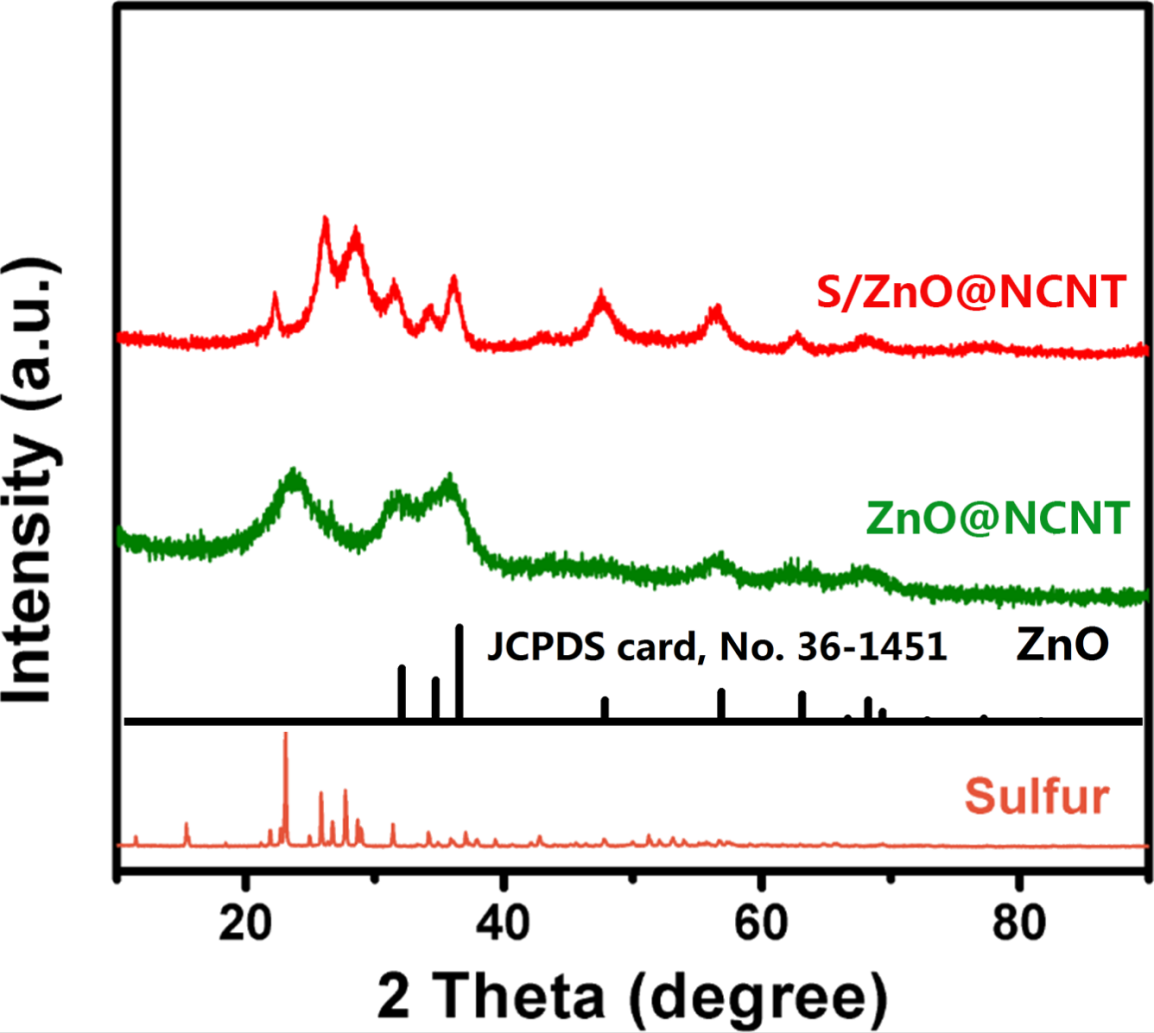
XRD patterns of S, ZnO@NCNT and S/ZnO@NCNT composite.

Figure 1 shows the XRD patterns of sulfur, ZnO@NCNT and S/ZnO@NCNT composite. It can be seen that S is successfully incorporated into the composite. The ZnO patterns can be assigned to hexagonal wurtzite (JCPDS no. 36-1451). For the patterns of ZnO@NCNT and S/ZnO@NCNT, the peaks at about 31.1°, 34.4°, 36.3°, 47.5°, 56.6°, 62.8° and 68° correspond to ZnO [17], and the broad peaks at around 23.8° are associated with NCNT [18]. The size of the ZnO nanoparticles was calculated based on the (101) peak using the Scherrer equation [13,19] and was found to be around 6.2 nm.

**Figure 2:**
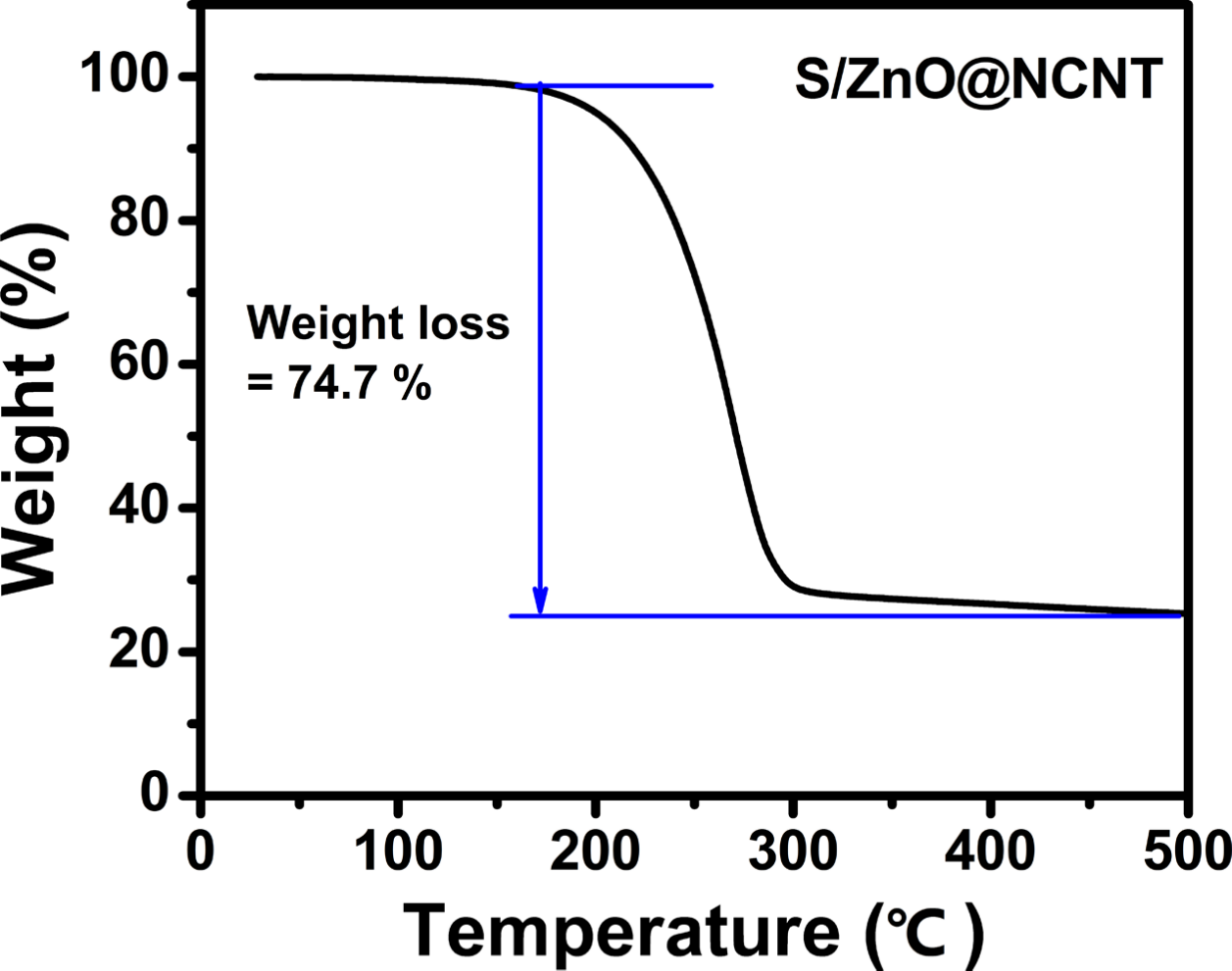
TGA curve of the S/ZnO@NCNT composite.

In order to determine the sulfur content in the S/ZnO@NCNT composite, the samples were studied by thermogravimetric analysis (TGA) in nitrogen gas. Figure 2 shows that the sulfur content in the S/ZnO@NCNT composite is about 74.7 wt %, which agrees well with the precursors proportions used during preparation. It can be concluded that the adopted technique, ball-milling followed by heat treatment, enables preparation of a high-performance composite of sulfur and ZnO@NCNT as it is shown in the following experiments.

**Figure 3:**
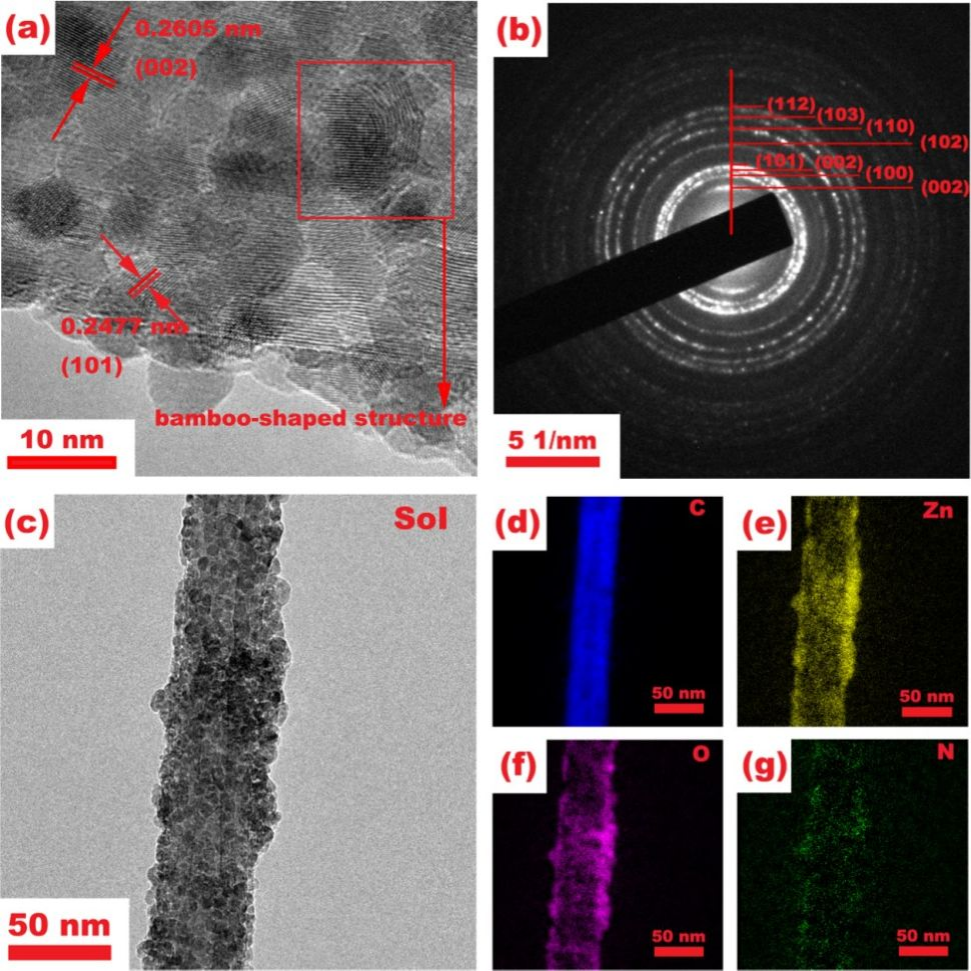
(a) HRTEM image; (b) SAED patterns; (c) TEM image; (d–g) EDX mapping images of the ZnO@NCNT composite.

Figure 3 illustrates the morphology and element distribution for the as-obtained ZnO@NCNT composite before S loading. ZnO@NCNT exhibits a bamboo-like shape, ZnO nanoparticles are uniformly coated on the NCNT walls, and most of the nanoparticles have a diameter of less than 10 nm (Figure 3c), which is consistent with the results of our previous research [13]. Energy-dispersive X-ray spectroscopy (EDX) also confirms the presence and even distribution of C, Zn, O and N in ZnO@NCNT (Figure 3d–g). The crystal lattice fringes with a *d*-spacing of 0.26 and 0.25 nm were observed in the HRTEM image of the ZnO@NCNT composite (Figure 3a), which correspond to the (002) and (101) planes of ZnO, respectively. Figure 3b shows the selected area electron diffraction (SAED) patterns of the ZnO@NCNT composite. The diffraction rings represent different planes of ZnO, revealing the polycrystalline structure of the as-prepared ZnO@NCNT. Morphology and structure of the ZnO@NCNT before S loading are similar to those in our previous studies, and were discussed in our previous work [13].

**Figure 4:**
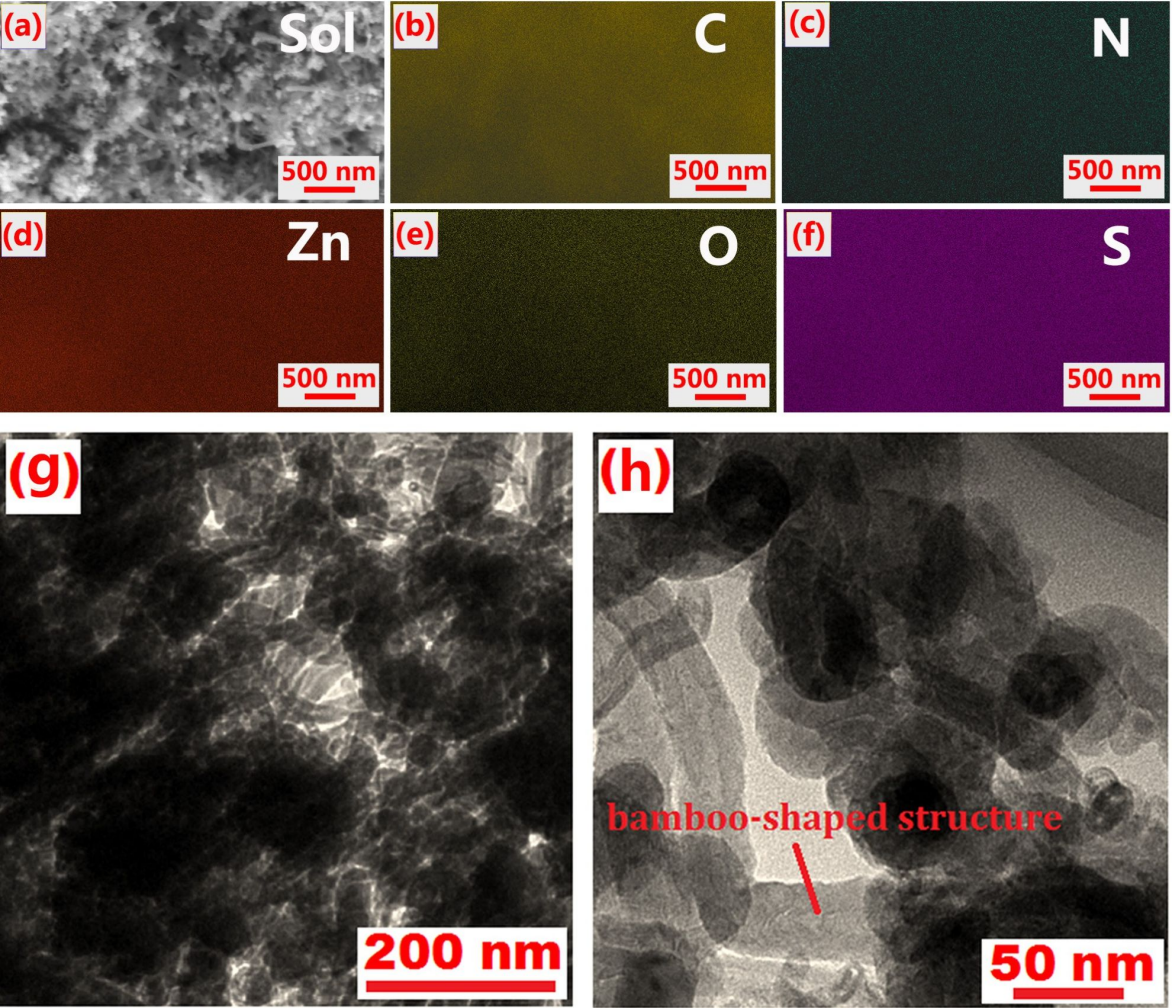
(a) SEM image; (b–f) EDX mapping; (g,h) TEM images of S/ZnO@NCNT composite.

After loading with S, the morphology of resulting S/ZnO@NCNT composite was again characterized by SEM and TEM (Figure 4). These images show that, although there are some large ZnO particles, the NCNT walls are coated by fine ZnO particles. Moreover, the EDX mapping confirms the successful loading and homogeneous distribution of S in the composite (Figure 4f). As can be seen from Figure 4g, the S/ZnO@NCNT composite still maintains a bamboo-shaped structure, but the material is agglomerated and cross-linked to each other, facilitating the transport of ions.

**Figure 5:**
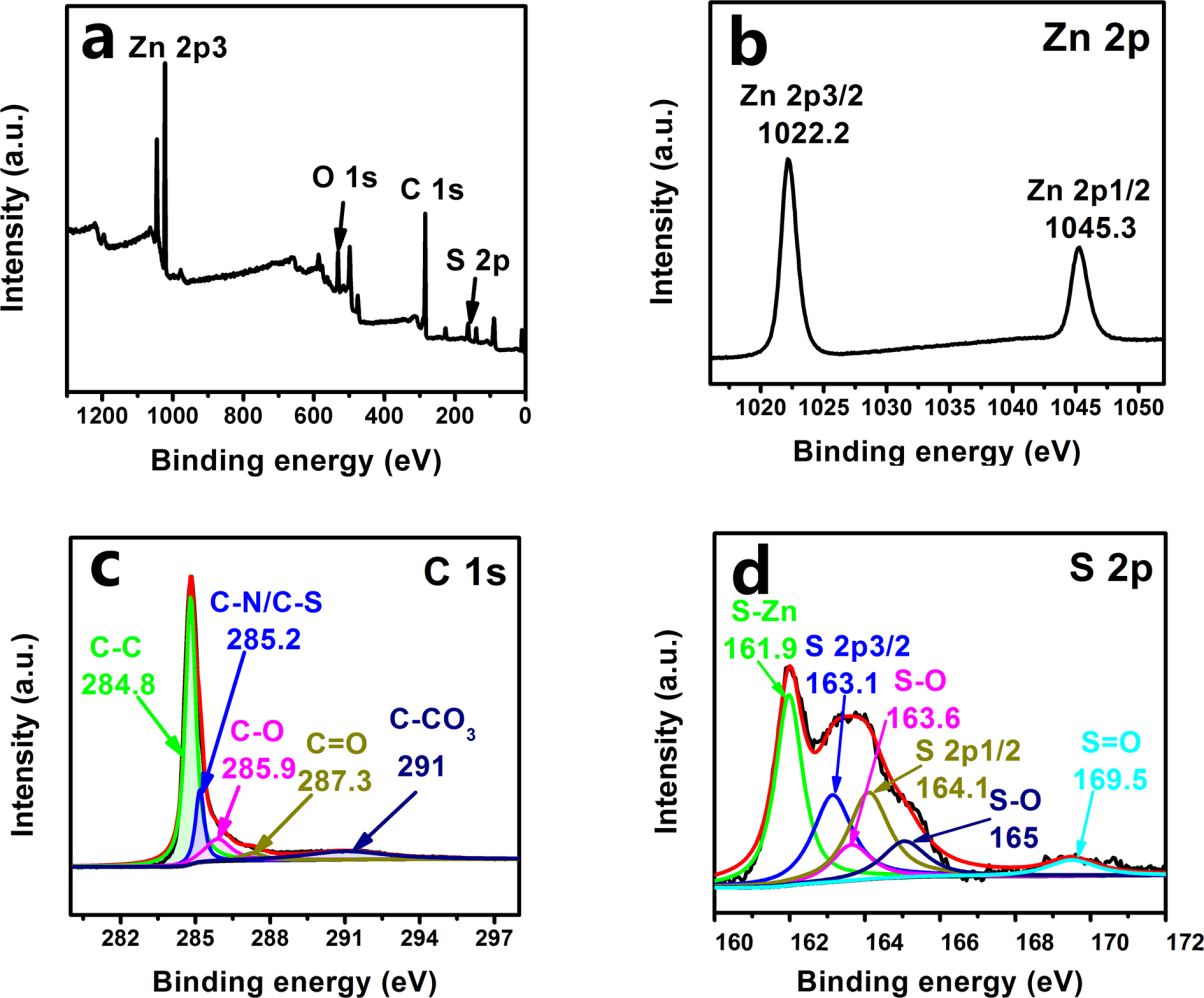
XPS spectra of S/ZnO@NCNT composite.

Some of the ZnO planes in the SAED patterns (Figure 3b), e.g., (101) and (112), are non-polar surfaces. They have been reported to have a much higher surface energy and therefore to be more active than the polar (100) plane [20]. Moreover, the high-Miller-index surface (103) observed in SAED pattern is usually more active than a low-Miller-index surface [21]. Therefore, one can expect that these active ZnO surfaces in the ZnO@NCNT composite can exhibit a strong bonding capacity for S. This will reduce the S losses to the electrolyte, and thus improve the cycling performance of the Li/S battery. In fact, the S–Zn and S–O bonds were confirmed by X-ray photoelectron spectroscopy (XPS) of the as-obtained S/ZnO@NCNT composite (Figure 5). In the S 2p spectrum, one major peak located at 161.9 eV actually corresponds to the S 2p in ZnS (Figure 5d) [22]. This suggests that, after S loading, S–Zn bonds were formed. The other two major peaks located at 163.1 and 164.1 eV in the S 2p spectrum could be identified as S 2p_3/2_ and S 2p_1/2_ species. Moreover, two weak peaks at 163.6 and 165 eV are associated with S–O bonds, and another weak peak located around 169.5 eV can be attributed to S=O bonds, which might result from S oxidation or S–O bonding on the ZnO surface [23]. An obvious C–N/C–S bonding was found at 285.2 eV in the C 1s spectrum (Figure 5c) [24]. Regarding the Zn 2p spectrum, the peaks located at 1022.2 and 1045.3 eV are the 2p_3/2_ and 2p_1/2_ states, respectively (Figure 5b) [25]. Since Zn–O and Zn–S have a similar XPS bonding energy in the two Zn states, they are not distinguishable in the Zn 2p spectrum.

**Figure 6:**
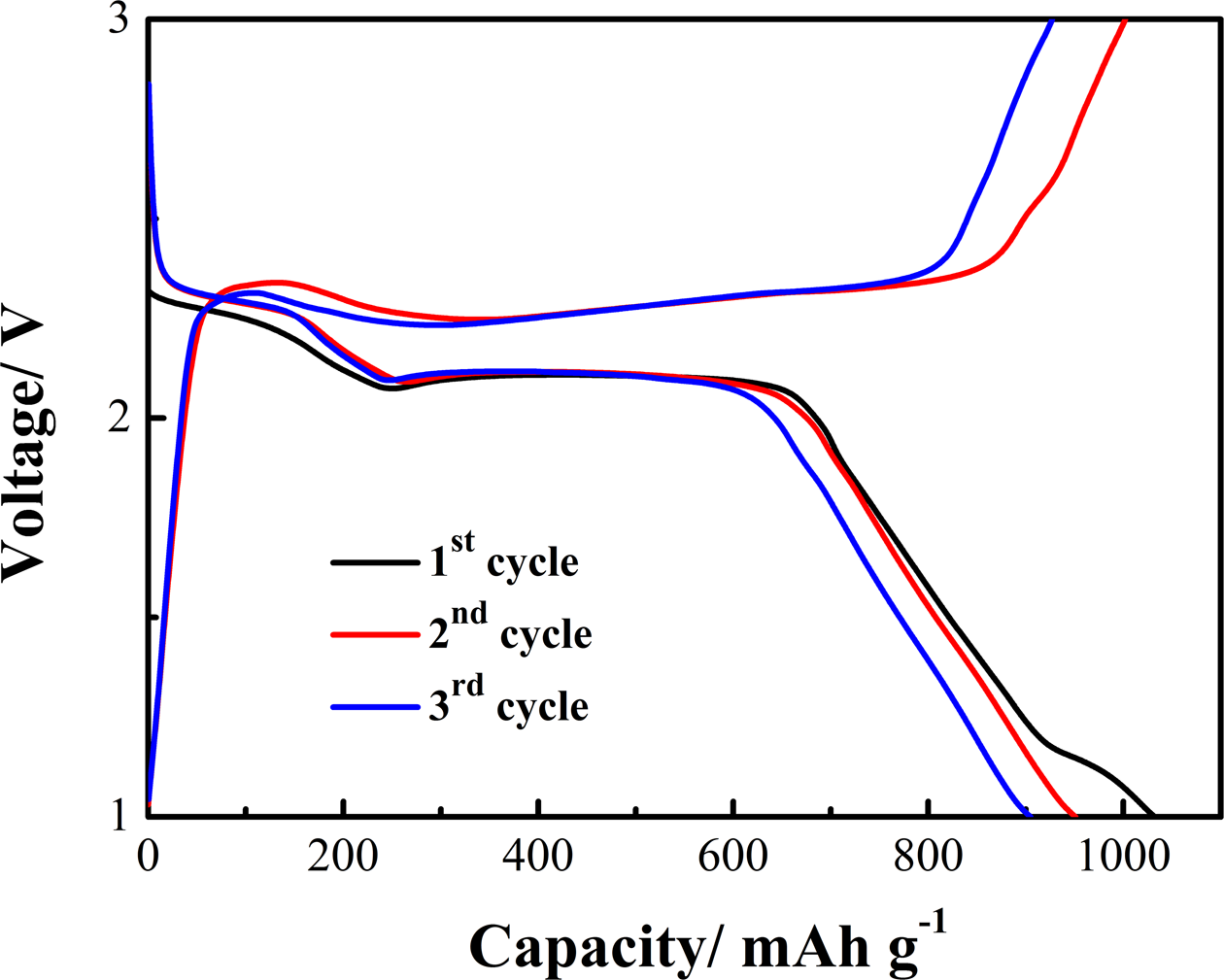
Discharge/charge voltage profiles of the S/ZnO@NCNT cathode for the initial three cycles at 0.2C.

The performance of the as-prepared S/ZnO@NCNT composite as a cathode in Li/S batteries was evaluated in lithium half-cell configuration. Figure 6 shows the discharge/charge potential profiles at 0.2C. Two typical plateaus appear during the discharge process. The plateau at 2.35 V can be related to the formation of long-chain polysulfides (Li_2_S*_n_*, *n* ≥ 4), another plateau at 2.1 V is associated with the electrochemical transition of Li_2_S*_n_* to lithium sulfide (Li_2_S) [26]. The S/ZnO@NCNT composite delivers an initial specific discharge capacity of 1032 mAh·g^−1^. There is a slight capacity fading and it drops to 905 mAh·g^−1^ in the third cycle. The potential plateaus were maintained in the first three cycles.

**Figure 7:**
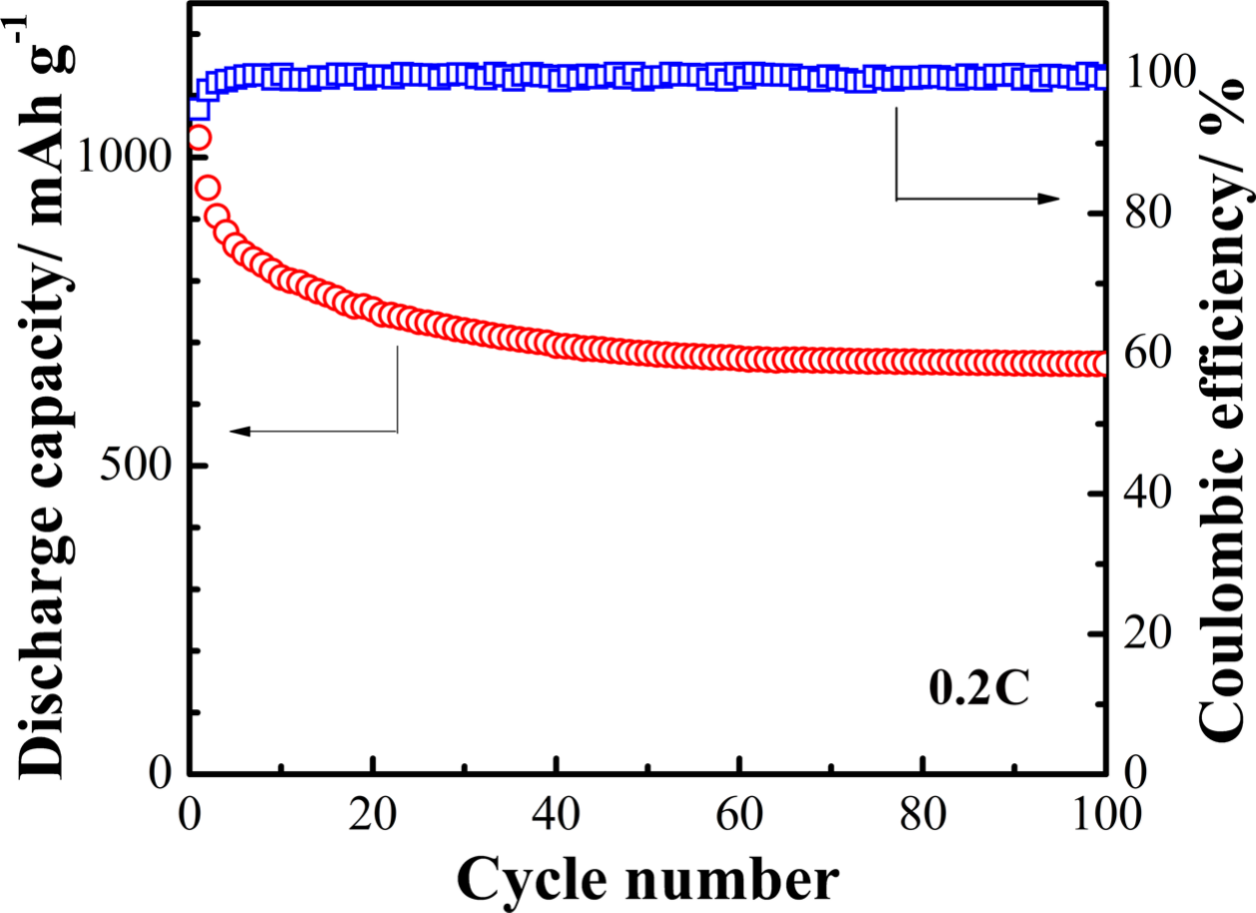
Cycling performance of the S/ZnO@NCNT cathode at 0.2C.

**Figure 8:**
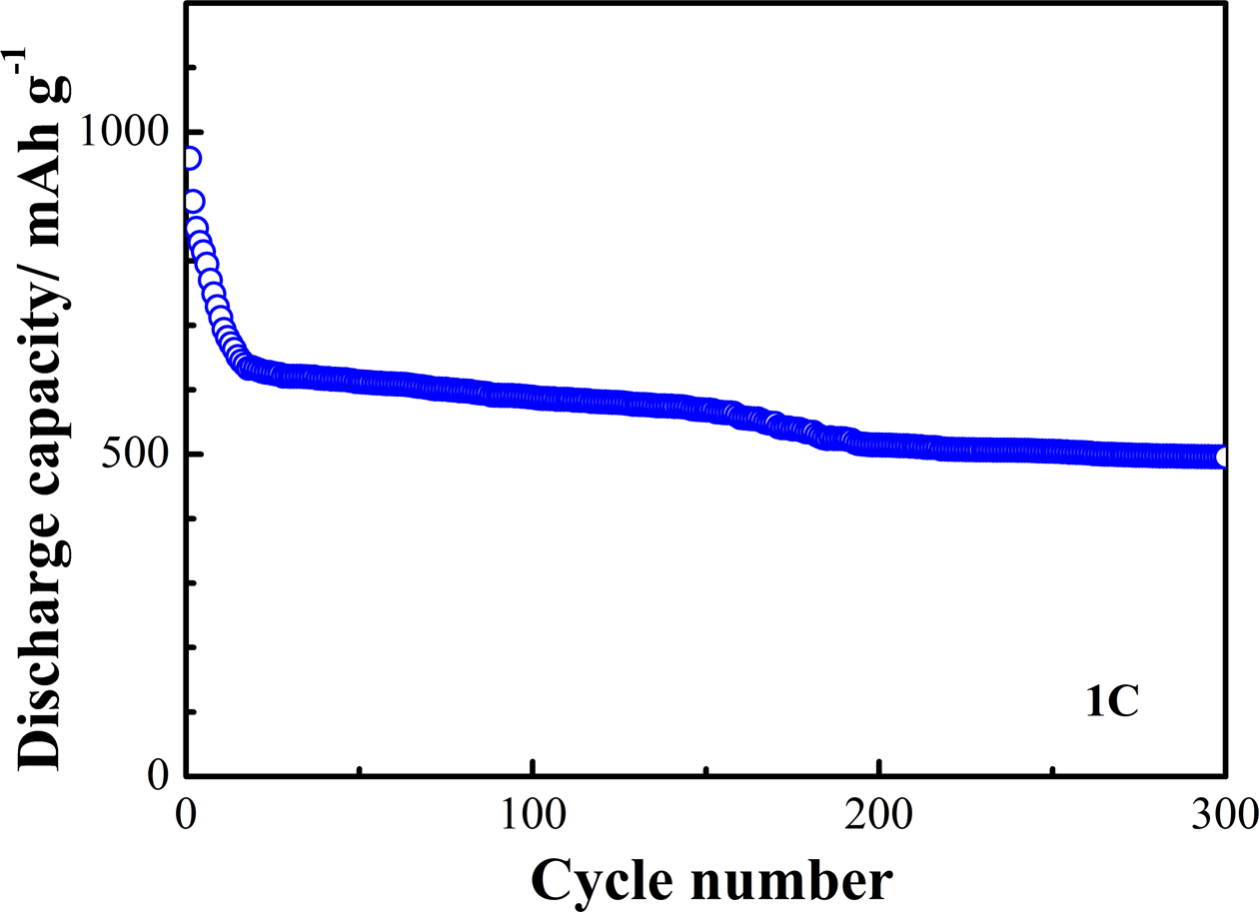
Long-term cycle life of the S/ZnO@NCNT cathode at 1C.

The cycling performance of the S/ZnO@NCNT cathode is presented in Figure 7. The results show that after 100 cycles the cathode still maintains a discharge capacity of 665 mAh·g^−1^. The coulombic efficiency of the S/ZnO@NCNT cathode remains unchanged above 99% after the 100th cycle, i.e., the S/ZnO@NCNT cathode exhibits very stable cycling performance. The long-term cycling behavior of the S/ZnO@NCNT cathode is presented in Figure 8. One can see that the system exhibits an extremely steady performance even after 300 cycles at 1C.

It is suggested that such a good performance originates from a strong bonding capacity of the reactive ZnO planes in the composite. As confirmed by the XPS analysis, the exposed active surfaces on ZnO can “hold” a large amount of S species through Zn–S and O–S bonds and therefore contribute to the observed excellent cycling performance.

**Figure 9:**
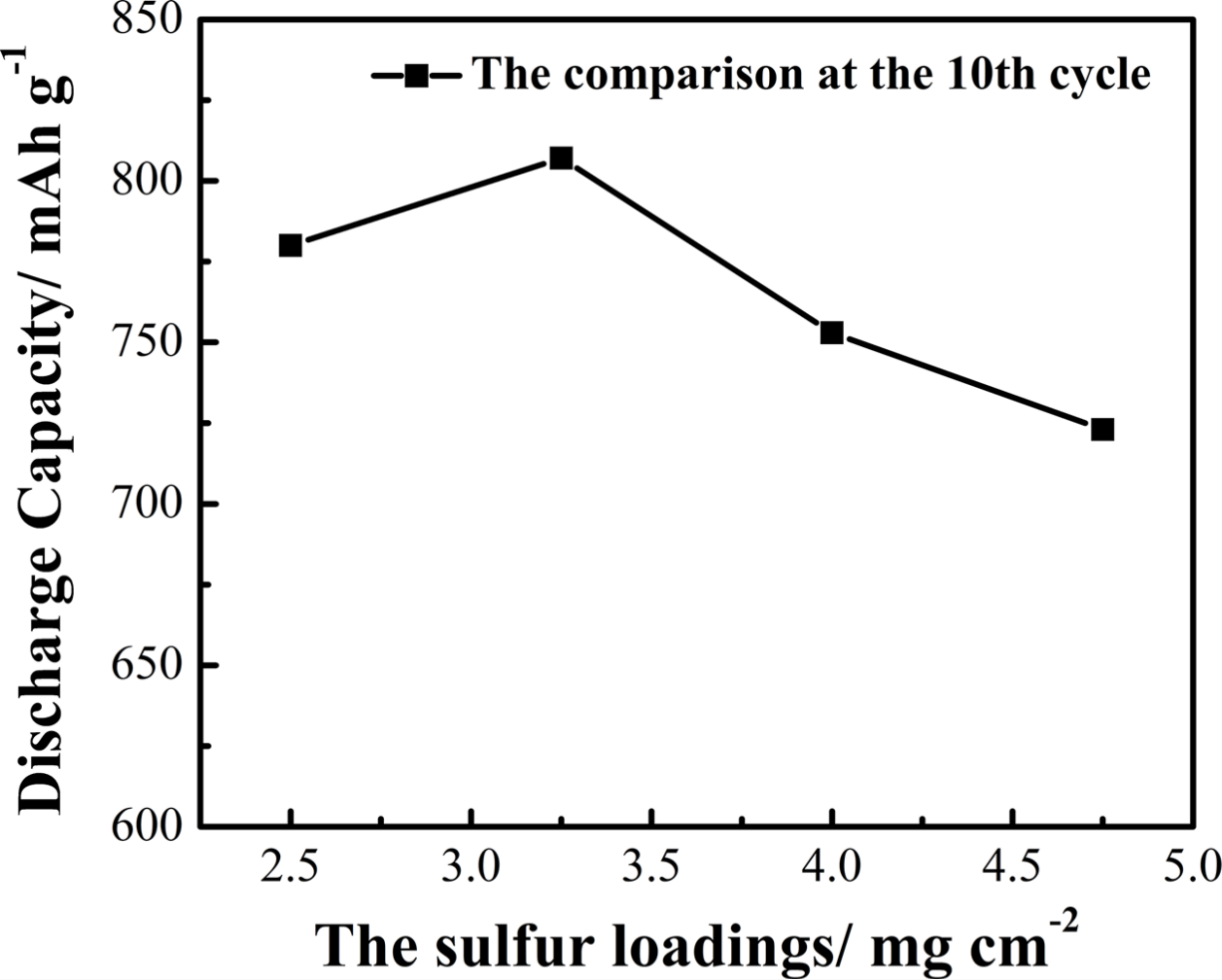
The performance comparison of S/ZnO@NCNT electrodes with sulfur loadings of 2.5, 3.25, 4.0 and 4.75 mg·cm^−2^ at the 10th cycle at 0.2C.

In a Li/S cell, the amount of sulfur loading is critical and strongly influences its electrochemical performance. Therefore, we fabricated S/ZnO@NCNT electrodes with various S loadings of about 2.50, 3.25, 4.00 and 4.75 mg·cm^−2^, and investigated the effect of loading on the discharge capacity of the composite. Figure 9 shows the comparison of discharge capacity at the tenth cycle at a current density of 0.2C for the samples with different S loading. It can be seen that the discharge capacity increases initially, and a high value of 805 mAh·g^−1^ was delivered after ten cycles at a S loading of 3.25 mg·cm^−2^. However, as the loading increased further, the capacity value reduced significantly. For example, at a high S loading of 4.75 mg·cm^−2^, the material could deliver a capacity of 723 mAh·g^−1^. This capacity decrease could be due to a reduced conductivity of the electrodes related to the excessive content of S. Furthermore, sulfur tends to agglomerate, and this negatively affects the cycle performance of the cell as well.

**Figure 10:**
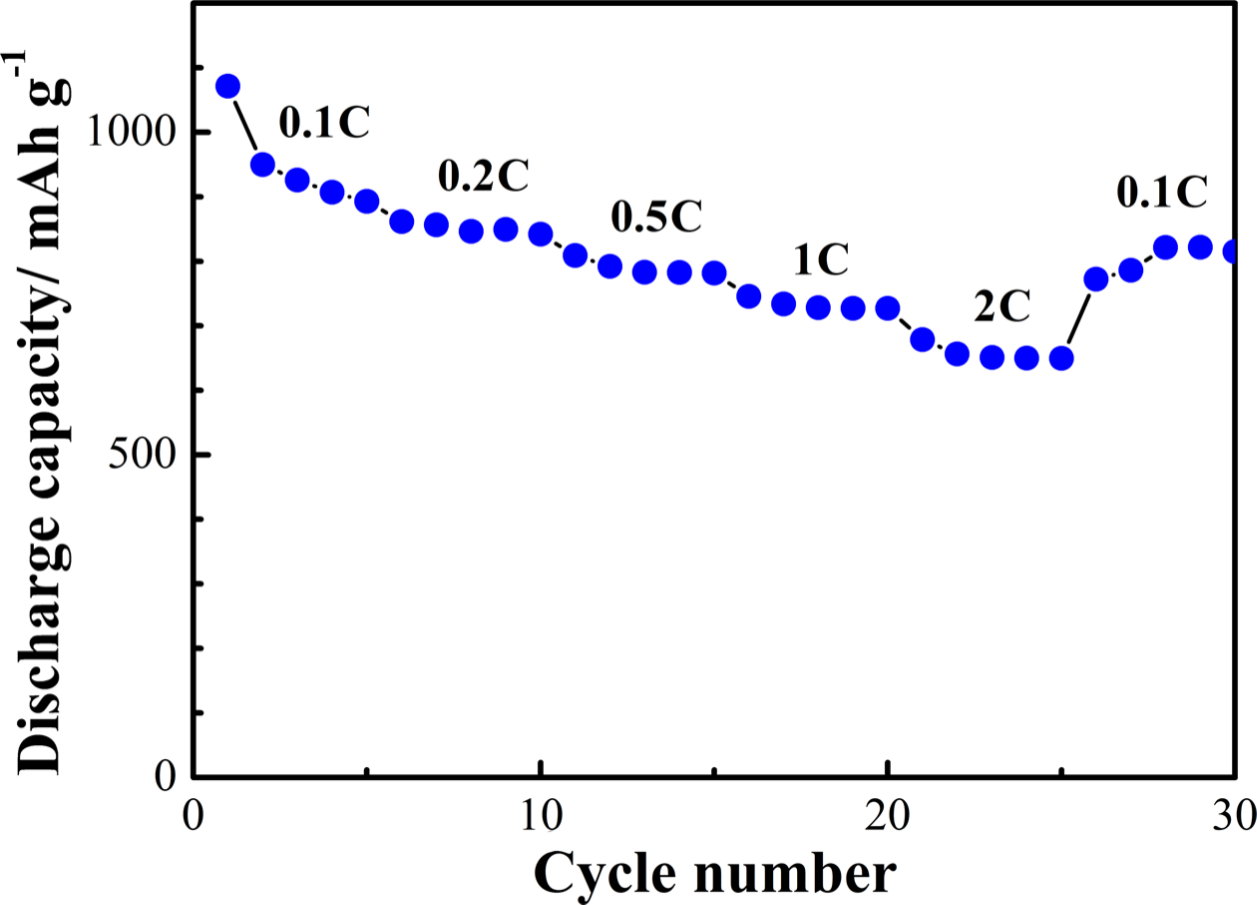
Rate capability of the S/ZnO@NCNT composite cathode.

**Figure 11:**
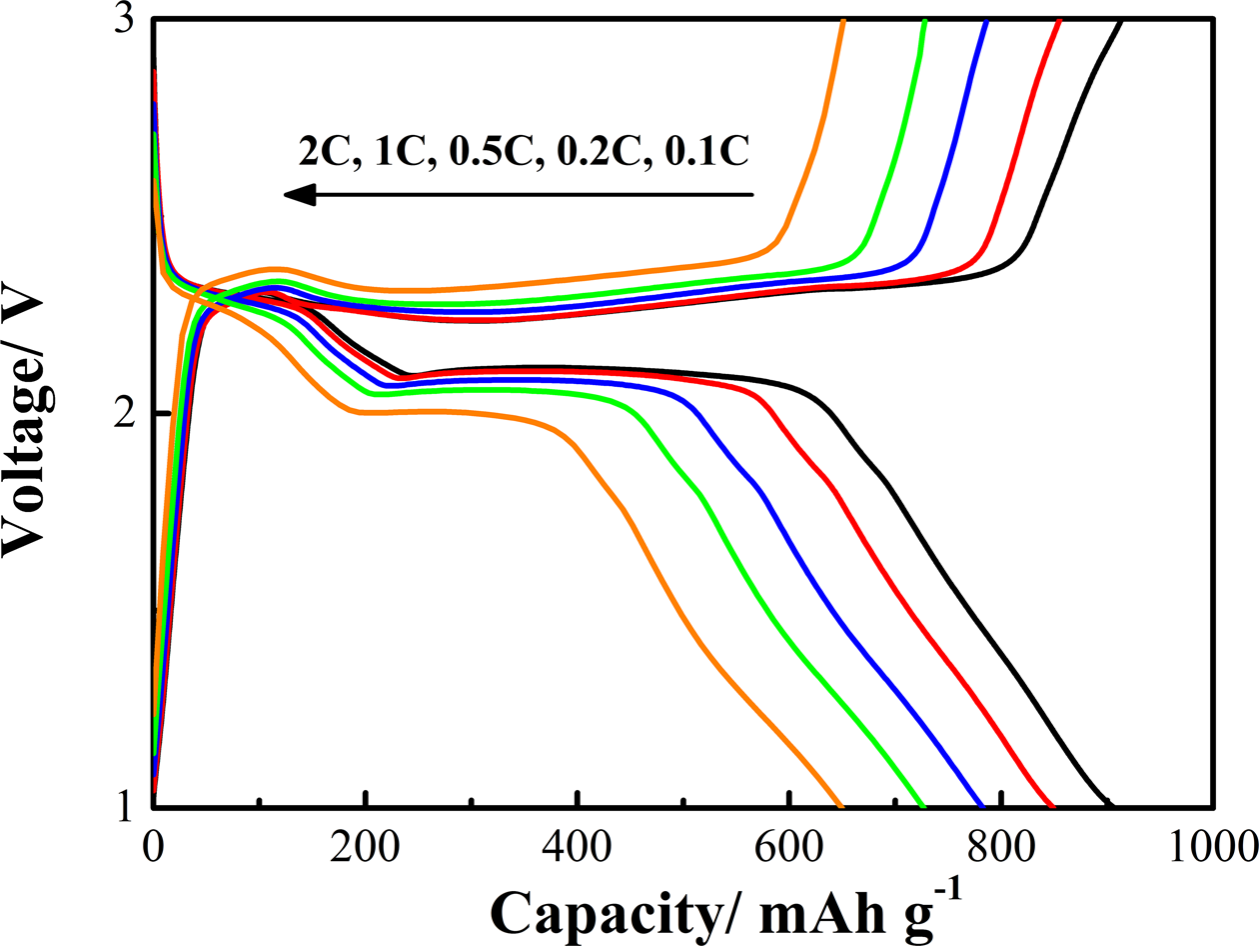
Discharge/charge voltage profiles of S/ZnO@NCNT composite cathode at various rates.

The rate capability of the S/ZnO@NCNT cathode at rates from 0.1C to 2C was studied as well (Figure 10). Although the discharge capacity of the S/ZnO@NCNT cathode gradually decreases with the cycling rate, at each individual rate from 0.2C to 2C, the composite cathode exhibit a relatively steady reversible capacity. At 2C, a reversible capacity of 650 mAh·g^−1^ was reached. When the current rate was changed back to 0.1C, the capacity of the S/ZnO@NCNT cathode recovered to 822 mAh·g^−1^. This indicates that the as-prepared S/ZnO@NCNT composite is very stable and can tolerate the abusive condition of high-rate Li ion insertion and deletion. In addition to a strong S “confinement” effect of the active ZnO surface, this might also be attributed to the NCNT network and the small size of ZnO nanoparticles (6.2 nm) in the composite, which both enhanced charge transfer and conductivity. Along with this, the NCNT network provides a large micro-scaffold in the S/ZnO@NCNT composite to accommodate S and Li ions, and, therefore, to buffer the volume expansion/shrinkage caused by the fast Li insertion/deletion. Moreover, small size ZnO nanoparticles provide abundant active sites for Li ion insertion and deletion and can also “buffer” the structural damage of the composite. In our previous work [13], it has been demonstrated that NCNT network and ZnO nanoparticles in ZnO@NCNT have these advantageous properties for the use in Li-ion batteries. The discharge/charge potential profiles of the cell at various rates also confirm the excellent stability of the S/ZnO@NCNT composite cathode (Figure 11). The results show that there is only a slightly fading of the potential plateaus with increasing rate. More importantly, it can be observed that there is only a small polarization of the electrode, which demonstrates highly reversible features of Li ion insertion/deletion in the S/ZnO@NCNT composite at various cycling rates.

Table 1 compares the performance data reported for Li/S batteries with the results of this work. The S/ZnO@NCNT electrode prepared in this work displays superior electrochemical performance, even with the higher sulfur loadings. The S/INC composite reported in our previous research work is based on INC, which has a high specific surface area and a large number of mesopores. Unlike ZnO@NCNT reported in the current work, which binds sulfur to the surface, INC encapsulates sulfur inside the pore structure and therefore provides a higher specific capacity. However, INC does not have ZnO-rich active sites and is prone to damage upon the intercalation/deintercalation of Li ions, so the cycle performance of this system is not as good as that of S/ZnO@NCNT. Therefore, the results of this study demonstrate that the NCNT core and the smaller size of the ZnO nanoparticles are effective to remarkably improve the electrochemical performance of the S/ZnO@NCNT electrode.

**Table 1:** Performance comparison of different electrodes for Li/S batteries.

material	reversible capacity (mAh·g^−1^)	cycle number	current density	applied potential range (V)	sulfur loading (mg·cm^−2^)	reference

MWNT@ZIF-S	380	25	0.1C	1.0–3.0	0.6	[27]
Al-ZnO@C/S	544	300	0.5C	1.8–2.6	3.3	[28]
S/PPy	503	100	0.1C	1.5–3	4	[26]
Fe_2_O_3_/S	442.3	100	0.5C	1.0–3.0	0.56	[29]
A-TiO_2-x_NSs-S	610	100	0.1C	1.7–2.8	1.17	[30]
Meso-C/S	470.2	300	0.5C	1.7–2.7	2.0	[31]
S/NGC	572	100	0.2C	1.7–2.8	3.4	[32]
S/INC	702	50	0.1C	1.0–3.0	3.0	[33]
S/ZnO@NCNT	665	100	0.2C	1.0–3.0	3.25	this work

## Conclusion

Nitrogen-doped carbon nanotubes coated with zinc oxide (ZnO@NCNT) were successfully prepared via a sol–gel synthetic route. They exhibit a unique ability to absorb polysulfides and, thus, to improve electrochemical properties of a S cathode. The S/ZnO@NCNT cathode has shown excellent cycling stability and rate capability in Li/S batteries. This enhanced electrochemical performance originates from its active ZnO surfaces, which can provide a strong bonding capability for S atoms. Moreover, it is believed that the large micro-scaffold in the NCNT network not only improves the conductivity of the composite, but also facilitates the modulation of S and Li ions, buffering the volume expansion/shrinkage caused by the fast Li insertion/deletion.

## Experimental

### Preparation of ZnO@NCNT composite

The ZnO@NCNT composite was synthesized by sol–gel synthesis [13,34]. In a typical synthesis, 7.717 g zinc acetate (Zn(CH_3_COO)_2_, ≥99%) was dissolved in 260 mL ethanol (C_2_H_5_OH, ≥99.7%). Meanwhile, 1.508 g lithium hydroxide (LiOH·H_2_O, ≥90%) was dissolved in another 260 mL of ethanol. The molar ratio of Zn(CH_3_COO)_2_ and LiOH·H_2_O was 1.3:1. These solutions were magnetically stirred until the reagents were completely dissolved. After that, the LiOH and Zn(CH_3_COO)_2_ solutions were mixed together and stirred for 20 min. Then, 0.17 g of nitrogen-doped multi-walled carbon nanotubes (NCNT, N content of 2.98%, Beijing Dk Nano Technology) was added to the above mixture solution under magnetic stirring for a week. The resulting black sol product was centrifuged and washed several times with deionized water and ethanol, then dried in a vacuum oven at 70 °C for 12 h to obtain the ZnO@NCNT composite.

### Preparation of S/ZnO@NCNT composite

The as-prepared ZnO@NCNT was mixed with nano-sulfur in a molar ratio of 1:3 by ball-milling at 350 min^−1^ for 3 h to obtain the sulfur composite precursor. The S/ZnO@NCNT composite was obtained by heating the precursor at 155 °C for 10 h, in argon flow with a heating rate of 5 °C·min^−1^. The sulfur-doping method was described in our previous study [33].

### Materials characterization

Powder X-ray diffraction (XRD, SmartLab, Rigaku Corporation) with Cu Ka radiation was used to analyze the crystal structure of the S/ZnO@NCNT sample. The chemical status and elemental compositions of the sample were investigated by X-ray photoelectron spectroscopy (XPS, Shimadzu Axis Ultra). Scanning electron microscopy (SEM) images were collected on a Hitachi S4800 scanning electron microscope. High-resolution transmission electron microscopy (HRTEM) images were recorded with a JEOL JEM-2100F transmission electron microscope. The elements distribution images were detected by using TEM at 160 kV. Thermogravimetric analysis (TA Universal Analysis 2000, SDT2960) was conducted from room temperature to 500 °C with a heating rate of 10 °C·min^−1^ in nitrogen.

### Electrochemical measurements

The cathode was fabricated by mixing 80 wt % as-prepared S/ZnO@NCNT composite, 10 wt % acetylene black and 10 wt % polyvinylidene fluoride (PVDF) in *N*-methyl-2-pyrrolidone (NMP). The resulting homogeneous slurry was coated on nickel foam and subsequently dried at 75 °C overnight. Metallic lithium foil served as a counter and reference electrode, and micro-porous polypropylene film (Cellgard 2300) was used as a separator. The electrolyte was 1 M lithium bistrifluoromethanesulfonamide (LiTFSI) in tetraethylene glycol dimethyl ether as a solvent. The CR2025 coin cells assembly was carried out in an argon-filled glovebox (Mikrouna, Shanghai). The charge/discharge cycling performances was investigated using a battery testing system (Neware, Shenzhen) in the potential range of 1–3 V vs Li/Li^+^.

## References

[1] Yuan G, Zhao Y, Jin H, Bakenov Z (2016). Ionics.

[2] Zhang Y, Zhao Y, Konarov A, Gosselink D, Soboleski H G, Chen P (2013). J Power Sources.

[3] Zhang Z, Jing H-K, Liu S, Li G-R, Gao X-P (2015). J Mater Chem A.

[4] Sun Q, Yadegari H, Banis M N, Liu J, Xiao B, Wang B, Lawes S, Li X, Li R, Sun X (2015). Nano Energy.

[5] Qiu Y, Li W, Zhao W, Li G, Hou Y, Liu M, Zhou L, Ye F, Li H, Wei Z (2014). Nano Lett.

[6] Xiao Z, Yang Z, Wang L, Nie H, Zhong M, Lai Q, Xu X, Zhang L, Huang S (2015). Adv Mater.

[7] Gopi C V V M, Venkataharitha M, Lee Y-S, Kim H-J (2016). J Mater Chem A.

[8] Rehman S, Tang T, Ali Z, Huang X, Hou Y (2017). Small.

[9] Rehman S, Guo S, Hou Y (2016). Adv Mater.

[10] Li Y, Cai Q, Lei W, Li Q, Xiang P, Gao B, Huo K, Chu P K (2016). ACS Appl Mater Interfaces.

[11] Li Z, Zhang J, Lou X W (2015). Angew Chem, Int Ed.

[12] Zhou W, Xiao X, Cai M, Yang L (2014). Nano Lett.

[13] Li H, Liu Z, Yang S, Zhao Y, Feng Y, Bakenov Z, Zhang C, Yin F (2017). Materials.

[14] Zhang Y, Wei Y, Li H, Zhao Y, Yin F, Wang X (2016). Mater Lett.

[15] Li H, Wei Y, Zhang Y, Yin F, Zhang C, Wang G, Zhumabay B (2016). Ionics.

[16] Zhao Y, Yin F, Zhang Y, Zhang C, Mentbayeva A, Umirov N, Hong X, Zhumabay B (2015). Nanoscale Res Lett.

[17] Zhang J, Gu P, Xu J, Xue H, Pang H (2016). Nanoscale.

[18] Le A V, Wang M, Shi Y, Noelle D, Qiao Y, Lu W (2015). J Appl Phys.

[19] Gandhi R R, Gowri S, Suresh J, Sundrarajan M (2013). J Mater Sci Technol.

[20] Meyer B, Marx D (2003). Phys Rev B.

[21] Brill G, Chen Y, Dhar N K, Singh R (2003). J Electron Mater.

[22] Siriwardane R V, Poston J A (1990). Appl Surf Sci.

[23] Fantauzzi M, Elsener B, Atzei D, Rigoldi A, Rossi A (2015). RSC Adv.

[24] Moon J, An J, Sim U, Cho S-P, Kang J-H, Chung C, Seo J H, Lee J, Nam K T, Hong B H (2014). Adv Mater.

[25] Chen C, Wang L, Li F, Ling L (2014). Mater Chem Phys.

[26] Yin F, Liu X, Zhang Y, Zhao Y, Menbayeva A, Bakenov Z, Wang X (2017). Solid State Sci.

[27] Yue Y, Guo B, Qiao Z A, Fulvio P F, Chen J, Binder A J, Tian C, Dai S (2014). Microporous Mesoporous Mater.

[28] Kong Y, Luo J, Jin C, Yuan H, Sheng O, Zhang L, Fang C, Zhang W, Huang H, Xia Y (2018). Nano Res.

[29] Zhao C, Shen C, Xin F, Sun Z, Han W (2014). Mater Lett.

[30] Wang H-C, Fan C-Y, Zeng Y-P, Zhang X-H, Li W-H, Liu S-Y, Sun H-Z, Zhang J-P, Sun L-N, Wu X-L (2017). Chem – Eur J.

[31] Bao W, Su D, Zhang W, Guo X, Wang G (2016). Adv Funct Mater.

[32] Vinayan B P, Diemant T, Lin X-M, Cambaz M A, Golla-Schindler U, Kaiser U, Behm R J, Fichtner M (2016). Adv Mater Interfaces.

[33] Li H, Wang Z, Zhang Y, Wang X, Zhao Y, Maximov M Y, Ji P, Yin F (2016). Russ J Appl Chem.

[34] Li H, Wei Y, Zhang Y, Zhang C, Wang G, Zhao Y, Yin F, Bakenov Z (2016). Ceram Int.

